# 1,5-Dimethyl-4-[(*E*)-3-phenoxy­benzyl­ideneamino]-2-phenyl-1*H*-pyrazol-3(2*H*)-one

**DOI:** 10.1107/S1600536808024409

**Published:** 2008-08-06

**Authors:** Yi-Feng Sun, Feng-Yu Zhang, Yang Liu, Zi-Ying Wang, Xue-Li Cheng

**Affiliations:** a Department of Chemistry, Taishan University, 271021 Taian, Shandong, People’s Republic of China; b Library, Taishan University, 271021 Taian, Shandong, People’s Republic of China

## Abstract

The title Schiff base, C_24_H_21_N_3_O_2_, adopts an *E* configuration with respect to the central C=N bond. The pyrazole ring and the central benzene ring attached to the imino group are almost coplanar. The phenyl ring attached to the pyrazole unit is twisted by 39.3 (2)° with respect to the pyrazole ring plane. The phen­oxy benzene ring makes a dihedral angle of 79.8 (2)° with the central benzene ring.

## Related literature

For related crystal structures, see: Sun *et al.* (2007*a* ▶,*b* ▶,*c* ▶).
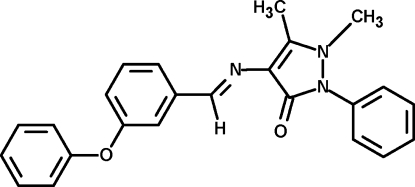


## Experimental

### 

#### Crystal data

C_24_H_21_N_3_O_2_
*M* *_r_* = 383.44
Triclinic, 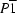

*a* = 7.6640 (12) Å
*b* = 8.3593 (14) Å
*c* = 16.731 (3) Å
α = 77.396 (3)°
β = 77.587 (2)°
γ = 76.240 (3)°
*V* = 1000.9 (3) Å^3^
*Z* = 2
Mo *K*α radiation
μ = 0.08 mm^−1^
*T* = 273 (2) K
0.18 × 0.16 × 0.12 mm

#### Data collection

Bruker SMART CCD area-detector diffractometer
Absorption correction: multi-scan (*SADABS*; Sheldrick, 1996 ▶) *T*_min_ = 0.985, *T*_max_ = 0.990
5300 measured reflections
3505 independent reflections
2584 reflections with *I* > 2σ(*I*)
*R*_int_ = 0.015

#### Refinement

*R*[*F* ^2^ > 2σ(*F* ^2^)] = 0.043
*wR*(*F* ^2^) = 0.117
*S* = 1.04
3505 reflections
264 parameters
H-atom parameters constrained
Δρ_max_ = 0.17 e Å^−3^
Δρ_min_ = −0.18 e Å^−3^

### 

Data collection: *SMART* (Bruker, 1997 ▶); cell refinement: *SAINT* (Bruker, 1997 ▶); data reduction: *SAINT*; program(s) used to solve structure: *SHELXS97* (Sheldrick, 2008 ▶); program(s) used to refine structure: *SHELXL97* (Sheldrick, 2008 ▶); molecular graphics: *SHELXTL* (Sheldrick, 2008 ▶); software used to prepare material for publication: *SHELXTL*.

## Supplementary Material

Crystal structure: contains datablocks global, I. DOI: 10.1107/S1600536808024409/fj2143sup1.cif

Structure factors: contains datablocks I. DOI: 10.1107/S1600536808024409/fj2143Isup2.hkl

Additional supplementary materials:  crystallographic information; 3D view; checkCIF report

## Figures and Tables

**Fig. 1.**
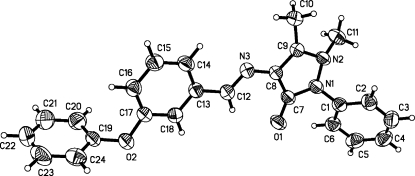
View of the molecule of (I) showing the atom-labelling scheme. Displacement ellipsoids are drawn at the 50% probability level. H atoms are shown as small spheres of arbitrary radii.

## supplementary crystallographic information

### Comment

Antipyrine (2,3-dimethyl-1-phenylpyrazol-5-one) and its derivatives have been long known for their wide spectrum of biological activities. As part of our ongoing studies of antipyrine derivatives, the title compound, (I), has been prepared and its crystal structure is reported here (Fig. 1).

The molecule adopts an E configuration with respect to the central C=N double bond (Fig. 1).The pyrazole ring (N1/N2/C7—C9), the C13—C18 phenyl ring and the imino group are almost coplanar which allows conjugation. But the C1—C6 phenyl ring is twisted with respect to the central pyrazole ring plane by 39.3 (2)°. In addition, the mean planes of the C13—C18 and C19—C24 phenyl rings make a dihedral angle of 79.8 (2)°. Therefore the molecule is not planar. The bond distances and angles agree with the corresponding values found in similar compounds (Sun *et al.*, 2007*a*,b,c).

### Experimental

A mixture of 4-aminoantipyrine (1 mmol) and 3-phenoxybenzaldehyde (1 mmol) in anhydrous ethanol (20 ml) was refluxed for 3 hr, and then cooled to room temperature. After cooling, the solvent was removed under reduced pressure and the solid residue was recrystallized from ethanol to yield the pure product(66% yield). m.p. 425–427 K. A single-crystal suitable for an X-ray structural analysis was obtained by slowly evaporating a ethanolic solution at room temperature.

### Refinement

All H atoms were initially located in a difference Fourier map. The methyl H atoms were then constrained to an ideal geometry with C—H distances of 0.96 Å and *U*_iso_(H) = 1.5*U*_eq_(C). All other H atoms were placed in geometrically idealized positions and constrained to ride on their parent atoms with C—H distances 0.93 Å and *U*_iso_(H) = 1.2*U*_eq_(C).

### Crystal data

**Table:**

C_24_H_21_N_3_O_2_	*Z* = 2
*M**_r_* = 383.44	*F*_000_ = 404
Triclinic, *P*1	*D*_x_ = 1.272 Mg m^−^^3^
Hall symbol: -P 1	Mo *K*α radiation λ = 0.71073 Å
*a* = 7.6640 (12) Å	Cell parameters from 1817 reflections
*b* = 8.3593 (14) Å	θ = 2–25.1º
*c* = 16.731 (3) Å	µ = 0.08 mm^−^^1^
α = 77.396 (3)º	*T* = 273 (2) K
β = 77.587 (2)º	Block, colorless
γ = 76.240 (3)º	0.18 × 0.16 × 0.12 mm
*V* = 1000.9 (3) Å^3^	

### Data collection

**Table:**

Bruker SMART CCD area-detector diffractometer	3505 independent reflections
Radiation source: fine-focus sealed tube	2584 reflections with *I* > 2σ(*I*)
Monochromator: graphite	*R*_int_ = 0.016
*T* = 273(2) K	θ_max_ = 25.1º
φ and ω scans	θ_min_ = 2.5º
Absorption correction: multi-scan(SADABS; Sheldrick, 1996)	*h* = −9→9
*T*_min_ = 0.985, *T*_max_ = 0.990	*k* = −9→8
5300 measured reflections	*l* = −19→19

### Refinement

**Table:**

Refinement on *F*^2^	Secondary atom site location: difference Fourier map
Least-squares matrix: full	Hydrogen site location: difference Fourier map
*R*[*F*^2^ > 2σ(*F*^2^)] = 0.043	H-atom parameters constrained
*wR*(*F*^2^) = 0.117	*w* = 1/[σ^2^(*F*_o_^2^) + (0.0553*P*)^2^ + 0.1379*P*] where *P* = (*F*_o_^2^ + 2*F*_c_^2^)/3
*S* = 1.04	(Δ/σ)_max_ = 0.001
3505 reflections	Δρ_max_ = 0.17 e Å^−^^3^
264 parameters	Δρ_min_ = −0.18 e Å^−^^3^
Primary atom site location: structure-invariant direct methods	Extinction correction: none

### Special details

**Table:**

Geometry. All e.s.d.'s (except the e.s.d. in the dihedral angle between two l.s. planes) are estimated using the full covariance matrix. The cell e.s.d.'s are taken into account individually in the estimation of e.s.d.'s in distances, angles and torsion angles; correlations between e.s.d.'s in cell parameters are only used when they are defined by crystal symmetry. An approximate (isotropic) treatment of cell e.s.d.'s is used for estimating e.s.d.'s involving l.s. planes.
Refinement. Refinement of *F*^2^ against ALL reflections. The weighted *R*-factor *wR* and goodness of fit *S* are based on *F*^2^, conventional *R*-factors *R* are based on *F*, with *F* set to zero for negative *F*^2^. The threshold expression of *F*^2^ > σ(*F*^2^) is used only for calculating *R*-factors(gt) *etc*. and is not relevant to the choice of reflections for refinement. *R*-factors based on *F*^2^ are statistically about twice as large as those based on *F*, and *R*- factors based on ALL data will be even larger.

### Fractional atomic coordinates and isotropic or equivalent isotropic displacement parameters (Å^2^)

**Table:**

	*x*	*y*	*z*	*U*_iso_*/*U*_eq_	
O1	−0.09698 (15)	0.55049 (16)	1.14179 (8)	0.0559 (3)	
O2	−0.41493 (16)	1.0621 (2)	0.79039 (8)	0.0727 (4)	
N1	0.17090 (17)	0.46344 (17)	1.19591 (8)	0.0451 (4)	
N2	0.34291 (17)	0.50949 (17)	1.17773 (9)	0.0436 (3)	
N3	0.15472 (18)	0.75399 (18)	1.00126 (8)	0.0463 (4)	
C1	0.1017 (2)	0.3971 (2)	1.27873 (10)	0.0436 (4)	
C2	0.1611 (2)	0.4293 (2)	1.34520 (11)	0.0534 (5)	
H2	0.2455	0.4978	1.3359	0.064*	
C3	0.0942 (3)	0.3590 (3)	1.42535 (12)	0.0644 (6)	
H3	0.1348	0.3798	1.4699	0.077*	
C4	−0.0311 (3)	0.2590 (3)	1.43974 (13)	0.0696 (6)	
H4	−0.0752	0.2118	1.4938	0.084*	
C5	−0.0915 (3)	0.2288 (3)	1.37361 (13)	0.0641 (5)	
H5	−0.1778	0.1620	1.3833	0.077*	
C6	−0.0253 (2)	0.2967 (2)	1.29318 (11)	0.0507 (4)	
H6	−0.0659	0.2749	1.2488	0.061*	
C7	0.0644 (2)	0.5536 (2)	1.13636 (10)	0.0430 (4)	
C8	0.1860 (2)	0.6450 (2)	1.07513 (10)	0.0412 (4)	
C9	0.3485 (2)	0.6120 (2)	1.10112 (10)	0.0431 (4)	
C10	0.5167 (2)	0.6735 (3)	1.05804 (11)	0.0573 (5)	
H10A	0.6070	0.5828	1.0386	0.086*	
H10B	0.5625	0.7175	1.0960	0.086*	
H10C	0.4894	0.7600	1.0117	0.086*	
C11	0.4972 (2)	0.3701 (2)	1.19262 (13)	0.0607 (5)	
H11A	0.5038	0.2889	1.1588	0.091*	
H11B	0.4801	0.3187	1.2502	0.091*	
H11C	0.6084	0.4116	1.1786	0.091*	
C12	0.0019 (2)	0.7838 (2)	0.97743 (10)	0.0477 (4)	
H12	−0.0906	0.7318	1.0099	0.057*	
C13	−0.0321 (2)	0.8980 (2)	0.90021 (10)	0.0442 (4)	
C14	0.1005 (2)	0.9784 (2)	0.84859 (11)	0.0502 (4)	
H14	0.2152	0.9607	0.8628	0.060*	
C15	0.0622 (2)	1.0844 (2)	0.77623 (12)	0.0548 (5)	
H15	0.1519	1.1379	0.7420	0.066*	
C16	−0.1063 (2)	1.1126 (2)	0.75369 (11)	0.0493 (4)	
H16	−0.1304	1.1832	0.7044	0.059*	
C17	−0.2385 (2)	1.0347 (2)	0.80524 (11)	0.0474 (4)	
C18	−0.2022 (2)	0.9280 (2)	0.87751 (11)	0.0492 (4)	
H18	−0.2927	0.8753	0.9115	0.059*	
C19	−0.4469 (2)	1.1295 (2)	0.71019 (11)	0.0482 (4)	
C20	−0.3803 (3)	1.0387 (3)	0.64795 (13)	0.0618 (5)	
H20	−0.3083	0.9324	0.6586	0.074*	
C21	−0.4186 (3)	1.1025 (3)	0.57065 (14)	0.0781 (7)	
H21	−0.3719	1.0402	0.5283	0.094*	
C22	−0.5239 (3)	1.2556 (4)	0.55469 (15)	0.0885 (8)	
H22	−0.5492	1.2985	0.5013	0.106*	
C23	−0.5939 (3)	1.3483 (3)	0.61630 (18)	0.0836 (7)	
H23	−0.6673	1.4536	0.6049	0.100*	
C24	−0.5555 (3)	1.2854 (2)	0.69626 (14)	0.0621 (5)	
H24	−0.6020	1.3472	0.7388	0.075*	

### Atomic displacement parameters (Å^2^)

**Table:**

	*U*^11^	*U*^22^	*U*^33^	*U*^12^	*U*^13^	*U*^23^
O1	0.0386 (7)	0.0680 (9)	0.0613 (8)	−0.0141 (6)	−0.0158 (6)	−0.0009 (6)
O2	0.0417 (7)	0.1136 (12)	0.0539 (8)	−0.0163 (7)	−0.0152 (6)	0.0112 (8)
N1	0.0369 (7)	0.0496 (9)	0.0479 (9)	−0.0084 (6)	−0.0113 (6)	−0.0030 (7)
N2	0.0319 (7)	0.0491 (8)	0.0488 (8)	−0.0050 (6)	−0.0119 (6)	−0.0051 (7)
N3	0.0424 (8)	0.0543 (9)	0.0427 (8)	−0.0071 (7)	−0.0114 (6)	−0.0085 (7)
C1	0.0398 (9)	0.0379 (9)	0.0476 (10)	0.0016 (7)	−0.0093 (7)	−0.0046 (7)
C2	0.0522 (10)	0.0498 (11)	0.0575 (12)	−0.0003 (9)	−0.0169 (9)	−0.0114 (9)
C3	0.0641 (12)	0.0728 (14)	0.0502 (12)	0.0075 (11)	−0.0153 (10)	−0.0160 (10)
C4	0.0636 (13)	0.0783 (15)	0.0484 (12)	0.0006 (12)	−0.0003 (10)	0.0024 (10)
C5	0.0561 (11)	0.0622 (13)	0.0640 (14)	−0.0119 (10)	0.0010 (10)	−0.0012 (10)
C6	0.0458 (10)	0.0500 (11)	0.0527 (11)	−0.0052 (8)	−0.0071 (8)	−0.0078 (8)
C7	0.0376 (9)	0.0453 (10)	0.0469 (10)	−0.0037 (7)	−0.0125 (7)	−0.0097 (8)
C8	0.0369 (8)	0.0476 (10)	0.0400 (9)	−0.0061 (7)	−0.0095 (7)	−0.0092 (7)
C9	0.0386 (9)	0.0492 (10)	0.0419 (9)	−0.0071 (7)	−0.0074 (7)	−0.0103 (8)
C10	0.0409 (10)	0.0769 (14)	0.0537 (11)	−0.0155 (9)	−0.0075 (8)	−0.0070 (10)
C11	0.0441 (10)	0.0598 (12)	0.0742 (13)	0.0031 (9)	−0.0205 (9)	−0.0080 (10)
C12	0.0426 (9)	0.0555 (11)	0.0445 (10)	−0.0105 (8)	−0.0102 (8)	−0.0044 (8)
C13	0.0422 (9)	0.0477 (10)	0.0437 (10)	−0.0073 (8)	−0.0109 (7)	−0.0089 (8)
C14	0.0398 (9)	0.0562 (11)	0.0568 (11)	−0.0123 (8)	−0.0131 (8)	−0.0070 (9)
C15	0.0497 (10)	0.0565 (12)	0.0590 (12)	−0.0211 (9)	−0.0095 (9)	−0.0004 (9)
C16	0.0508 (10)	0.0451 (10)	0.0497 (10)	−0.0113 (8)	−0.0128 (8)	0.0020 (8)
C17	0.0378 (9)	0.0568 (11)	0.0470 (10)	−0.0085 (8)	−0.0112 (8)	−0.0050 (8)
C18	0.0429 (9)	0.0589 (11)	0.0449 (10)	−0.0142 (8)	−0.0078 (8)	−0.0024 (8)
C19	0.0363 (9)	0.0573 (11)	0.0488 (11)	−0.0105 (8)	−0.0137 (8)	0.0028 (8)
C20	0.0598 (12)	0.0541 (12)	0.0644 (13)	−0.0029 (9)	−0.0083 (10)	−0.0070 (10)
C21	0.0711 (14)	0.1030 (19)	0.0636 (15)	−0.0168 (14)	−0.0149 (12)	−0.0192 (13)
C22	0.0640 (14)	0.129 (2)	0.0611 (15)	−0.0159 (15)	−0.0246 (12)	0.0161 (15)
C23	0.0583 (13)	0.0630 (14)	0.108 (2)	0.0043 (11)	−0.0239 (13)	0.0230 (14)
C24	0.0521 (11)	0.0516 (12)	0.0806 (15)	−0.0063 (9)	−0.0097 (10)	−0.0134 (10)

### Geometric parameters (Å, °)

**Table:**

O1—C7	1.2264 (19)	C11—H11A	0.9600
O2—C17	1.383 (2)	C11—H11B	0.9600
O2—C19	1.387 (2)	C11—H11C	0.9600
N1—C7	1.399 (2)	C12—C13	1.462 (2)
N1—N2	1.4130 (18)	C12—H12	0.9300
N1—C1	1.413 (2)	C13—C18	1.386 (2)
N2—C9	1.376 (2)	C13—C14	1.387 (2)
N2—C11	1.471 (2)	C14—C15	1.378 (2)
N3—C12	1.269 (2)	C14—H14	0.9300
N3—C8	1.395 (2)	C15—C16	1.376 (2)
C1—C6	1.382 (2)	C15—H15	0.9300
C1—C2	1.386 (2)	C16—C17	1.374 (2)
C2—C3	1.382 (3)	C16—H16	0.9300
C2—H2	0.9300	C17—C18	1.375 (2)
C3—C4	1.369 (3)	C18—H18	0.9300
C3—H3	0.9300	C19—C20	1.362 (3)
C4—C5	1.379 (3)	C19—C24	1.371 (3)
C4—H4	0.9300	C20—C21	1.353 (3)
C5—C6	1.379 (3)	C20—H20	0.9300
C5—H5	0.9300	C21—C22	1.346 (4)
C6—H6	0.9300	C21—H21	0.9300
C7—C8	1.445 (2)	C22—C23	1.366 (4)
C8—C9	1.354 (2)	C22—H22	0.9300
C9—C10	1.483 (2)	C23—C24	1.393 (3)
C10—H10A	0.9600	C23—H23	0.9300
C10—H10B	0.9600	C24—H24	0.9300
C10—H10C	0.9600		
			
C17—O2—C19	118.13 (13)	H11A—C11—H11B	109.5
C7—N1—N2	110.26 (13)	N2—C11—H11C	109.5
C7—N1—C1	125.01 (13)	H11A—C11—H11C	109.5
N2—N1—C1	119.24 (13)	H11B—C11—H11C	109.5
C9—N2—N1	105.45 (12)	N3—C12—C13	121.58 (17)
C9—N2—C11	119.28 (14)	N3—C12—H12	119.2
N1—N2—C11	114.71 (14)	C13—C12—H12	119.2
C12—N3—C8	121.20 (15)	C18—C13—C14	118.58 (16)
C6—C1—C2	119.78 (17)	C18—C13—C12	119.30 (16)
C6—C1—N1	119.10 (15)	C14—C13—C12	122.11 (15)
C2—C1—N1	121.11 (16)	C15—C14—C13	120.02 (16)
C3—C2—C1	119.65 (19)	C15—C14—H14	120.0
C3—C2—H2	120.2	C13—C14—H14	120.0
C1—C2—H2	120.2	C16—C15—C14	121.13 (17)
C4—C3—C2	120.64 (19)	C16—C15—H15	119.4
C4—C3—H3	119.7	C14—C15—H15	119.4
C2—C3—H3	119.7	C17—C16—C15	118.93 (17)
C3—C4—C5	119.6 (2)	C17—C16—H16	120.5
C3—C4—H4	120.2	C15—C16—H16	120.5
C5—C4—H4	120.2	C16—C17—C18	120.61 (16)
C6—C5—C4	120.6 (2)	C16—C17—O2	123.25 (16)
C6—C5—H5	119.7	C18—C17—O2	116.10 (16)
C4—C5—H5	119.7	C17—C18—C13	120.72 (17)
C5—C6—C1	119.70 (18)	C17—C18—H18	119.6
C5—C6—H6	120.1	C13—C18—H18	119.6
C1—C6—H6	120.1	C20—C19—C24	120.94 (18)
O1—C7—N1	123.73 (16)	C20—C19—O2	120.53 (17)
O1—C7—C8	131.93 (16)	C24—C19—O2	118.42 (17)
N1—C7—C8	104.32 (14)	C21—C20—C19	120.2 (2)
C9—C8—N3	121.71 (15)	C21—C20—H20	119.9
C9—C8—C7	108.38 (15)	C19—C20—H20	119.9
N3—C8—C7	129.89 (14)	C22—C21—C20	120.4 (2)
C8—C9—N2	111.07 (15)	C22—C21—H21	119.8
C8—C9—C10	128.06 (16)	C20—C21—H21	119.8
N2—C9—C10	120.86 (14)	C21—C22—C23	120.5 (2)
C9—C10—H10A	109.5	C21—C22—H22	119.8
C9—C10—H10B	109.5	C23—C22—H22	119.8
H10A—C10—H10B	109.5	C22—C23—C24	120.1 (2)
C9—C10—H10C	109.5	C22—C23—H23	120.0
H10A—C10—H10C	109.5	C24—C23—H23	120.0
H10B—C10—H10C	109.5	C19—C24—C23	117.9 (2)
N2—C11—H11A	109.5	C19—C24—H24	121.1
N2—C11—H11B	109.5	C23—C24—H24	121.1
